# Ethnographic Qualitative Study to Explore the Sociocultural Values, Nutritional Potential, and Health Benefits of Dabi Teff ( *Eragrostis Tef* ) Grown in Western Ethiopia

**DOI:** 10.1002/fsn3.71130

**Published:** 2025-10-25

**Authors:** Diriba Chewaka Tura

**Affiliations:** ^1^ Department of Human Nutrition Wollega University Nekemte Ethiopia

## Abstract

The rural Ethiopian populations have a rich traditional knowledge and practices linked to cereals as food and medicine (nutraceutical). This study aimed to explore and document the sociocultural values, nutritional potential, health benefits, and cultivation trends of dabi teff ( *Eragrostis tef* ) grown in Western Ethiopia. An exploratory qualitative study design was employed through focus group discussions and key informant interviews. Primary data were collected from purposively selected participants using a semi‐structured questionnaire. Data were analyzed manually; transcribed verbatim, thoroughly read, color‐coded, categorized into themes, translated, and reported. Three major themes emerged. The results show that there are many sociocultural values about dabi teff long‐rooted in the community. There is a well‐recognized nutritional potential and health benefits of dabi teff (prized as medicine). After eating the different food forms from dabi teff, particularly the mooqa manyee, it increases blood volume, boosts energy, and repairs/strengthens the backbone and fractured bones. There is ignorance in passing down the traditional practices. Dabi teff reaches maturity in a short time and is harvested twice within one rainy season, but its cultivation is declining compared to the past. The use of the crop as a complementary food is not common. The findings suggest that dabi teff could be used to address food and nutrition security beyond its ethnocultural significance. The sustainable production and utilization of such a valuable crop require integration of the study into national nutrition strategies and complementary feeding programs. Integration into agrobiodiversity conservation initiatives is also required to protect and safeguard our food system for the future.

## Graphical abstract

There is a life‐long practice of enjoying a number of food forms from dabi teff. Dabi teff has a promising nutritional potential and prized as a medicinal food. Dabi teff can be used in food formulation for nutritional enhancement and health benefits.

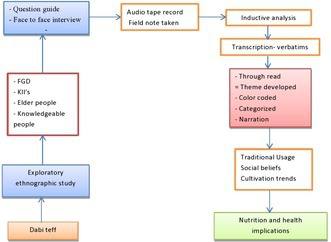

## 1 Introduction

Cereal crops occupy a remarkable and central position in ensuring food and nutrition security due to their high worldwide production, containing considerable amounts of energy, proteins, carbohydrates, minerals, vitamins, essential fatty acids, dietary fibers, and certain hormone precursors (Grivetti and Ogle 2000; Gibson et al. 2006). Despite such a substantial contribution to food and nutrition security, having medicinal value and sources of income generation, many crops across the world are underutilized and/or forgotten (Magbagbeola et al. 2010).

The sub‐Saharan African region is endowed with a rich diversity of underutilized and/or forgotten crops, where little or no attention was given in terms of research and development and policy framework that could promote their extensive agricultural production, marketing, industrial utilization, as well as home consumption (Magbagbeola et al. 2010).

The rural populations in Ethiopia have a rich indigenous knowledge concerning the use of food crops as food and medicine (Abebe and Ayehu 1993; Dechassa 2010). Such indigenous knowledge is applied to preserve food crops as a means of survival during times of food shortage in fighting against food insecurity challenges. Using traditional foods is still very common, especially in rural areas of developing countries, including Ethiopia, because there are elders and other knowledgeable community members who are the key sources of traditional knowledge (Dechassa 2010). However, this indigenous knowledge among the rural communities is affected by fragile traditional skills (older people replaced by younger) that are likely to be lost when people immigrate to towns or to other regions, and can be lost by lifestyle changes, the Westernization or industrialization, when young people ignore practicing and passing down the traditional practices in their community (Abebe and Ayehu 1993). Therefore, local and indigenous knowledge of traditional/indigenous foods should be carefully considered and documented, when possible, with the cooperation of indigenous and local populations, because such work is valued as a vital instrument in guiding future policies for agriculture and responding to the Sustainable Development Goals (SDGs) agenda to address food and nutrition security.

Teff ( *Eragrostis tef* ) is an ancient tropical super cereal food where both its origin and a major center of diversity are in Ethiopia (Ketema 1997), and it was first domesticated between 4000 and 1000 bc (Roseberg et al. 2005). Teff grain was reported to have been discovered in a pyramid that is thought to date back to 3359 bc (Dijkstra et al. 2008). According to the report by Ebba (1975), there are 34 different varieties/cultivars of teff identified in Ethiopia based on their morphological characteristics.

Teff is an important part of Ethiopia's cultural heritage and national identity. Nowadays, teff's international popularity is rapidly growing, and it was labeled as one of the latest super‐foods of the 21st century (Daba 2017). The use of teff grain as a foodstuff is already well known in some parts of the world, like the Netherlands, North America, Australia, Canada, South Africa, and Kenya, where its cultivation trial has shown a wide range of adaptation (Dijkstra et al. 2008; Ebba 1975; Gebremariam et al. 2014). Seeking to ensure food security for the Ethiopian population and to protect local markets from inflation or to keep domestic prices low, the export of non‐processed teff has been banned since 2006 (FAO 2013). Due to this, teff is mostly traded domestically, where more than 70% of the Ethiopian population uses teff as a traditional staple meal (Gebremariam et al. 2014).

Teff is primarily cultivated and used as a staple food in the western, northern, and central parts of Ethiopia, and it is the dominant cereal crop in terms of production. Until recently, 99% of the global teff production took place in Ethiopia, making the teff value chain localizable (Daba 2017). The common vernacular name of the crop is derived from the Amharic word “teffa”, which means “lost” due to the small size of the grain and how easily it is lost if dropped: *Taafi* (Oromiffa) and *Taf* (Tigrigna), which are the main languages of the Ethiopian people (Dijkstra et al. 2008). Teff grows in both drought‐stressed and water‐logged soil conditions, and it has a short growing season with low rainfall requirements of 450–550 mm and a temperature range of 10°C–27°C (Ebba 1975; Ketema 1997).

Over the past two decades, teff has become an issue of agronomic, nutritional, food technological, microbiological, chemical, and physical research programs (Dijkstra et al. 2008). It is highly nutritious, with its grain protein content (10%–12%) similar to other cereals (Daba 2017; Tura et al. 2023b). Teff protein is compared with egg protein and with an ideal protein for children between 2 and 5 years old. A lot of scholars have also demonstrated that teff starch has a low glycemic index (decreases the incidence of diabetes), which is the reason why this microscopic grain is initiating a big war between different grain producers and processors (Dijkstra et al. 2008).

Besides providing protein and calories, teff is a high source of minerals, such as calcium, zinc, magnesium, phosphorus, copper, aluminium, barium, thiamine, and particularly higher iron (Mengesha 1966; Tura et al. 2023a), which are the most limiting minerals in developing countries. The content of iron and calcium in teff is higher compared to other cereals such as wheat, barley, maize, and oats (Mengesha 1966; Tura et al. 2023a). It has an excellent amino acid composition, with lysine levels higher than wheat or barley (Mengesha 1966). Teff is also a gluten‐free crop and is gaining popularity as an alternative grain for people with gluten sensitivity, called celiac disease (Bultosa 2007), and this qualifies the crop as a medicinal food. Presently, the only effective treatment for celiac disease is a life‐long gluten‐free diet (Dijkstra et al. 2008). Teff also has many other health benefits, where most Ethiopians hardly suffer from diseases like anemia, osteoporosis, and diabetes (Gamboa and Ekris 2008). Teff may help in maintaining glycemic control and can be used for the management of diabetic diseases because teff consumption does not result in the rise of blood glucose levels due to its low glycemic index (Viljamaa et al. 2005).

Dabi teff, a typical farmer variety teff (landrace), is one of the teff varieties marginally cultivated or an underutilized/forgotten crop in Western Ethiopia. Dabi teff is the “afaan oromoo” language name for an early maturing variety of dark red teff. Farmers in Wollega and Illuababor, western Ethiopia, cultivate dabi teff either for its grain seeds or its straw. Dabi teff can grow under low rainfall requirements and this property, together with an early maturing time, makes the crop a unique variety of teff amenable to climate‐smart as well as nutrition‐sensitive agriculture. This signifies that dabi teff is one of the most important crops with considerable potential to support food and nutrition security in western Ethiopia. It can also be used for farm income generation. These properties of dabi teff could be used to address the SDGs, particularly to attain the zero hunger target, the nutritional well‐being and good health, and also for ensuring sustainable agriculture in Ethiopia. These are the rationales why dabi teff warrants scientific attention beyond its ethnocultural significance.

The key underlying cause of child malnutrition among households with low socioeconomic status is a lack of purchasing power for quality foods for their children, and they consume foods of low nutritive value. Price‐wise, the red teff varieties grown in Ethiopia, including dabi teff, are the cheapest. For example, Rashid and Negassa (2011) reported that the market price of mixed teff and red teff was 24% and 55% less than the white teff. This statement specifies that the price of dabi teff is quite affordable for all socioeconomic classes. In addition to lower prices, red teff varieties have been implicated in the low incidence of anemia in Ethiopia, which is presumed to be due to the grain's high iron content (Gebremariam et al. 2014). But paradoxically, according to the EPHI and ICF (2019) report, 57% of under‐five children in Ethiopia are anemic, which leads to the suspicion that there are low or nil practices of using red teff as an ingredient in complementary food preparation. It is also reported that preschool children in Africa, including Ethiopia, have some of the highest rates of anemia in the world, nearly 56% (United Nations) (Dijkstra et al. 2008).

There are many sociocultural values (traditional knowledge and practices), nutritional and health benefits surrounding dabi teff among rural elderly people in particular and the consumers in general. Yet, no attention was given in terms of research to explore and document the sociocultural values, the believed nutritional potential, and health benefits of dabi teff grown in western Ethiopia that could promote its extensive production and utilization. Thus, this study was aimed at exploring and documenting the sociocultural values, nutritional potential, health benefits, and the cultivation trends of dabi teff ( *Eragrostis tef* ) grown in Western Ethiopia.

## 2 Methods

### 2.1 Study Setting

**FIGURE 1:**
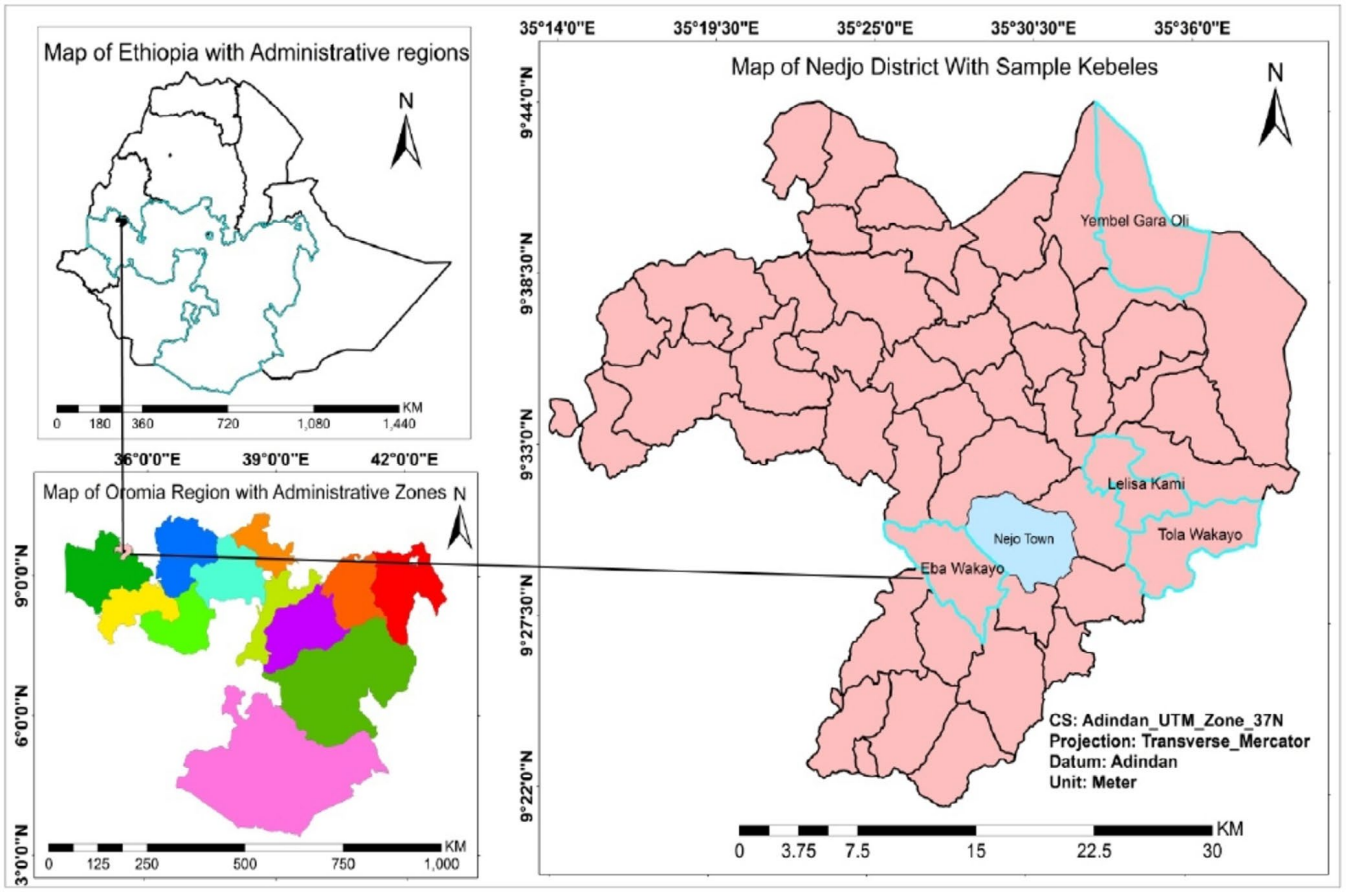
Map of Nedjo district with sampled kebeles, Ghimbi Zone, Western Ethiopia.

The study was conducted in Nedjo district (Figure 1), Ghimbi Zone, Western Ethiopia, which is located 575 km away to the west of Addis Ababa. There are 23 districts in the zone. According to the Ethiopian Central Statistical Agency (CSA), the zone has a total population of 1,350,415, of which 671,538 are men and 678,877 are women, and 92.42% of the population are rural inhabitants, with an area of 10,833.19 km^2^. The dominant means of livelihood in the zone is agriculture, cultivating cereals such as maize, barley, sorghum, teff, finger millet, oats, and legumes such as beans and peas, which are components of the major staple meals in the area. The zone is one of the major coffee‐growing areas, and it shares a huge total coffee output in Ethiopia.

### 2.2 Study Design

A community‐based exploratory qualitative study design was employed to elicit and collect deep ethnocultural information surrounding dabi teff in the community using a focus group discussion (FGD) and key informant interviews (KIIs). This design was used to best explore the sociocultural values (traditional knowledge and practices), nutritional potential, health benefits, and cultivation trends linked to dabi teff, and it worked well to uncover the way the traditional values are practiced and praised in the community.

### 2.3 Description of Dabi Teff

As described in the introduction section, the material used in this study is dabi teff, a local name for *Eragrostis tef*. It is a typical farmer variety of teff (landrace), which is one of the teff varieties marginally cultivated or underutilized/forgotten in Western Ethiopia. Dabi teff is the “afaan oromoo” language name for an early maturing variety of dark red teff. Farmers in Wollega and Illuababor, western Ethiopia, cultivate dabi teff either for its grain seeds or its straw. The contribution of dabi teff grains to household food and nutrition security is considerable when viewed through a food security lens. Dabi teff can grow under low rainfall requirements, and this property, together with an early maturing, makes the crop a unique variety of teff amenable to climate‐smart agriculture, where its ethnocultural practices are not yet studied.

### 2.4 Sampling Technique and Study Participants Selection Procedure

Selection of the study participants began with first visiting the zonal agricultural bureau to identify the potential dabi teff growing area in the zone where Nedjo district, with its sub‐districts, the kebeles (the smallest administrative unit), was identified. The district has 34 kebeles. Then, in consultation with the district administrators, four kebeles with a good history of growing dabi teff and also near the main road were purposively selected to arrive at the study participants. The names of the four selected kebeles were Eba waqayyo kebele, Tola Waqayyo kebele, Yambal kebele, and Lalisa kami kebele.

Male and female elders aged around 50 (the grandmothers/fathers) who were perceived to have good traditional knowledge and beliefs regarding dabi teff were purposely selected for the FGD, nominated by the agricultural development army (DA) in the village. The KIIs were comprised of the DA and knowledgeable community members who were also purposively selected.

The criteria for FGD eligibility were older males and females born and living (believed to have lived experience and perception) in the study area, able to understand and speak the local language and given their consent. The eligibility criterion for the composed KIIs was education level (college level), and they were believed to be knowledgeable and able to understand and speak the local language.

### 2.5 Data Collection Tools

An open‐ended semi‐structured FGD guide interview question was used for both the FGD and the KIIs (Supporting Information S1) to elicit and collect ethnocultural information or the sociocultural values, including traditional usage, social beliefs, and cultivation trends, its usage as complementary food, the storage quality, and some other qualities of dabi teff. Any new experiences arising from the participants were able to build on each other's ideas and were used for the enrichment of the information. One FGD session was conducted from each of the four purposively selected kebeles, comprising 8–10 participants. A total of four FGDs were conducted, which were assumed to be enough to capture more than 90% of the themes to be generated from our homogeneous study participants. A total of 38 FGD discussants participated. Concerning the KIIs, four key informants from each kebele were interviewed, including three knowledgeable/educated community members and one agricultural development army (DA) worker in the village. A total of 16 key informants participated. The number of FGDs and KIIs was determined based on the level of information saturation, which was established by transcribing the discussions of each day's session. The homogeneity of the respective FGD participants was maintained by sex. Male and female discussants were interviewed separately to freely express their ideas.

The guide questions were first prepared in the English language and translated into “afaan Oromoo” language by the researcher for easy communication and then translated back to the English language to confirm the consistency of the tool to generate qualitative original data.

### 2.6 Tool Validation and Pilot‐Testing

For the purpose of tool validation, three experts—two culture and tourism experts and one agricultural development army (DA)—were employed who were working in the district office. For the purpose of pilot testing, four potential participants (two male and two female) were employed. They were purposively selected knowledgeable people (similar to subjects of the final study), and they were interviewed two times from an area similar to the study area. The purpose of this pilot testing was to obtain feedback concerning the appropriateness, content validity, easy understanding of all the questions, and the estimated time required to complete the session. Finally, the data collection tool was reviewed and adjusted based on the feedback obtained. The data were analyzed manually using subsequent standard steps, where all the information collected was transcribed verbatim, reviewed, thoroughly read and internalized, color‐coded, categorized, and synthesized into themes, translated, and reported.

### 2.7 Data Collection Procedures

Data were collected in December 2024, and the discussion was facilitated by the principal researcher and two assistants trained before the actual data collection period, one cultural and tourism expert and one development army (DA) member having a tertiary level education and working in the study area. These research assistants did not have any relationship with the participants before the commencement of the study. The participants were gathered at village posts on non‐market and weekend days to avoid barriers to participation and the discussion was held face‐to‐face, where none of them declined to participate in the study.

The facilitators introduced themselves to the participants and warmly welcomed and invited them to introduce themselves because they were a group of strangers, and finally, a relationship was established between them. Then, after the objective of the discussion was briefed to them, followed by initiating the discussion session which was initiated one by one based on the guide using the local language. The information generated by each participant was audio‐taped using digital recorders and also by taking handwritten notes using a notebook with the help of the research assistants. This was done to ensure triangulation of the data with the record and/or refer to the notebook if the recorder fails. Further extensive probing was conducted to determine if there was any left/missing information. The FGD discussion took from 60 to 70 min while the KIIs took from 40 to 50 min. Redundancies of ideas were considered to be information saturation and were removed every evening during the transcription of the day's work and preliminary analysis. The study participants were further probed to reflect more deeply on the meaning of their comments, and new questions were added whenever an information gap was identified.

### 2.8 Trustworthiness of the Data

The quality of qualitative research is assured by meeting standards of trustworthiness through addressing credibility and transferability. To satisfy credibility, participants from different kebeles were included. Additionally, focus group discussions, key informant interviews, and field notes were used in the analysis of the data. The study provided descriptions of the setting, sampling procedure, eligibility criteria, tool validation and pilot testing, interview processes, and findings to strengthen the transferability to different areas. Validity and reliability were ensured by triangulation of the data gathered from the interviews with the information obtained at the FGD. Further, we have returned the transcribed text to some participants (member‐checks) for reviewing for any corrections and to ensure reliability.

### 2.9 Data Analysis

**FIGURE 2:**
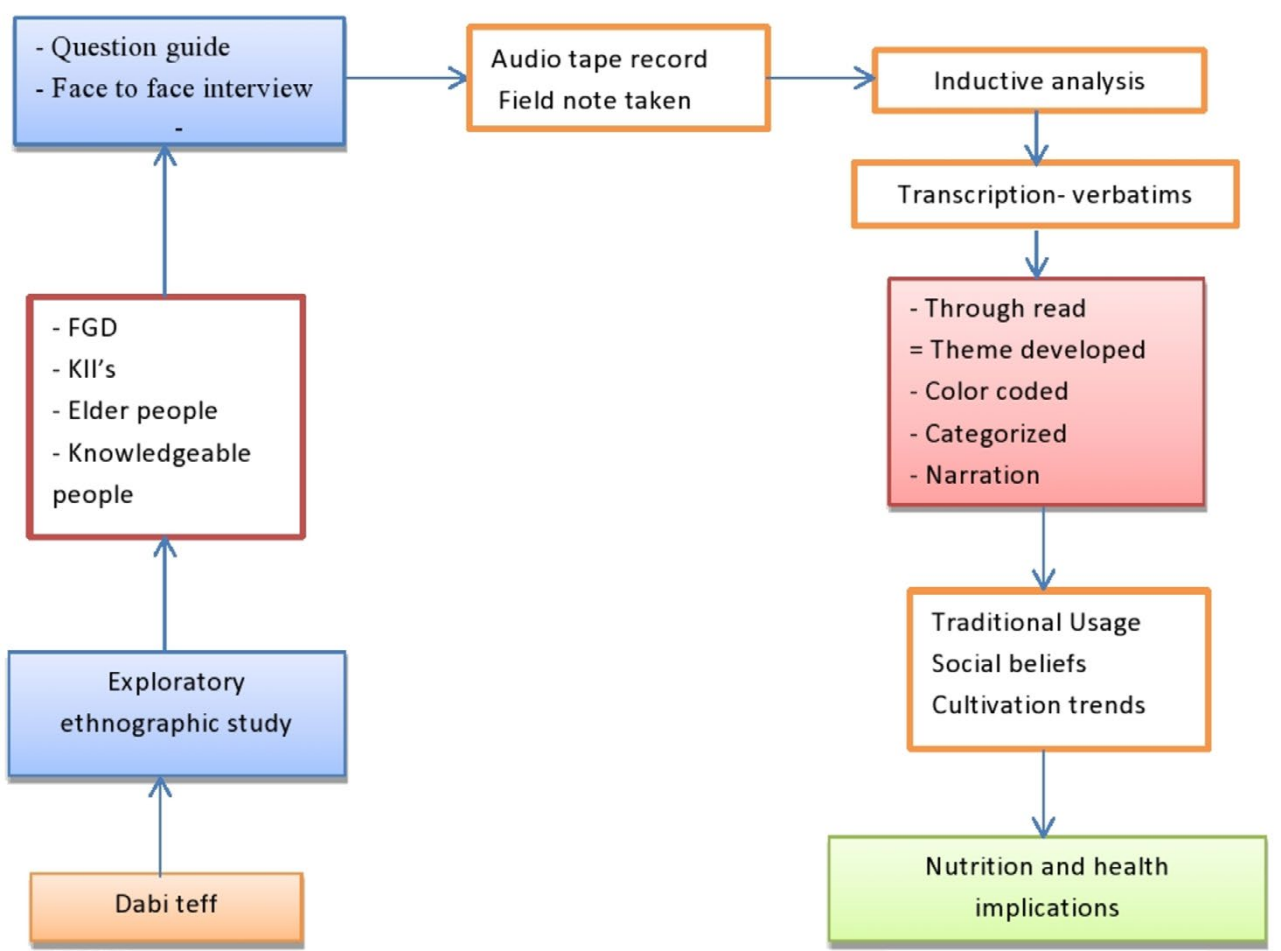
Graphical abstract.

An inductive approach was employed, and the collected data were analyzed manually using the guideline of the systematic text condensation lens (Eliot, A. and Associates 2005). The overall flow diagram to conduct and analyze the study was depicted by the graphical abstract (Figure 2). The principal researcher, together with the assistants (the research team), carefully listened to the audio record and reviewed the notebook. The information obtained was checked against the objectives, and finally, it was transcribed verbatim. The transcript was read thoroughly and repeatedly to grasp and internalize a thorough sense of the overall content in the texts, to identify central meaningful units in the transcribed material, and was reviewed. The contents were color‐coded. Major categories of information were captured and synthesized to develop themes. Any diverse cases linked to the codes, categories, and themes were corrected through team discussions, and a few were modified to enhance readability. Finally, the document was translated from the local language (afaan Oromoo) to the English language.

For presentation in the results section, summaries of the findings were condensed (texts with similar colors were pooled) and narrated under each of the identified themes or constructs and substantiated with powerful quotes as illustrations to persuade the claims. Steps in the conduct, analysis, and reporting of this study were arranged according to the guidelines outlined in the consolidated criteria for reporting qualitative research (COREQ) (Tong et al. 2007); all details are provided in the Supporting Information S2. The subsequent steps of the data analysis are presented in Table 1.

**TABLE 1:** Steps of the ethnographic qualitative data analysis, Nedjo district, Western Ethiopia, 2024.

Steps	Description
Transcription of data	The audiotape recorded was carefully listened and the handwritten notes were reviewed and transcribed verbatim (word perfect).
Internalizing the data	Grasping and internalizing of the whole information was achieved by thoroughly and repeatedly reading the transcript to get the real meaning of words from the participants and reflective notes that were taken.
Generating color codes	After reading thoroughly, similar texts were color‐coded line by line attempting to categorize major information.
Categorizing captured information	Major categories of information were captured (texts with similar coded colors were pooled together) to form potential themes
Synthesizing themes	The pooled texts were grouped together and themes were developed guided by the objectives followed by interpretation.
Reviewing themes	The themes were checked with the coded data extracts and assessed in terms of how well they represented the entire data set.
Defining and naming of themes	The names and definitions of each theme were refined, and a short description (short paragraph) of each theme was developed by the principal investigator.
Narration and producing a report	A completed report of the analysis was written by the principal researcher by narrating each of the emerged themes, substantiated with powerful quotes and relating to the original research question. Finally, the report was read and reviewed by all the authors.

## 3 Results

### 3.1 Background Characteristics of the Study Participants

A total of 54 participants were involved in this study. The FGD was comprised of 20 elderly males and 18 elderly females, while the KIIs comprised of 10 males and 6 females. They represent a wide age range (26–62 years), and the educational status ranged from no formal education to secondary level and above (Table 2).

**TABLE 2:** Sociodemographic characteristics of the study participants (*n* = 54), Nedjo district, Western Ethiopia, 2024.

Characteristics of participants	Categories	Frequency	Percent (%)
Age range	32–62	—	—
Sex	Male	30	55.56
	Female	24	44.44
Educational status	No formal education	20	37.04
	Primary	16	29.63
	Secondary and above	18	33.33
Occupational status	Employee	10	18.52
	Housewife/farmer	38	70.37
	Daily laborer	6	11.11

Reviewing, through reading and internalization of the transcripts, resulted in the emergence of three major themes that were synthesized into heading titles, followed by short paragraphs and triangulated or substantiated with powerful quotes from the participants.

The major themes that emerged were as follows:

- Traditional usage, including preparation schemes/protocols
- Social beliefs linked to dabi teff in the community
- Cultivation trends of dabi teff from the past to the present

### 3.2 Traditional Usage of Dabi Teff, Including Its Food Preparation Schemes

According to the information and experiences generated from the study participants, it was understood that there is a very similar traditional usage of dabi teff. People in western Ethiopia are highly connected with the traditional usage of dabi teff, where the participants had lifelong practices of preparing and enjoying different forms of favorite foods from dabi teff, which are highly praised and “liked very much” in the community.

All participants involved in the FGD stated as follows:

… many traditional food forms such as muke/mooqa (gruel), kita (sweet dry unleavened bread), cumboo, cafaqoo, cacabssa, cuukoo, unkuroo (the favorite cultural dishes), bread and injera/budena (pancake‐like local bread) can be prepared from dabi teff…

Further, the FGD participants described the special food form called mooqa manyee (gruel prepared from dabi teff and bull hooves), which was also supported by the key informant's interviewers. A 62‐year‐old FGD female participant described the preparation scheme of mooqa manyee as follows:

…mooqa manyee is a recipe prepared from bulls' hooves and dabi teff. First, the nails of the hooves would be removed using fire; then, other unwanted parts like the skin would be cleaned and it would be chopped at their joint points. Then, it would be cooked overnight, where its meat and bones would be separated apart. A gruel from either dabi teff alone or mixed with oats and/or barley flour would be prepared separately and the cooked hooves will be added to it, and then mixed together while it is on fire, adding other required ingredients (homemade spices like garlic and salt). The oromo people like to add what is called “amole chew” salt, not table salt, no butter is added to it, it will be mixed in a wider big pan/dish, called “sataate”, after which it would be served to a family member as a “muke” while it is hot….

The kita (sweet, dry unleavened bread) is usually consumed during breakfast and snack time. Fresh kita of dabi teff has a distinctive flavor smell that people sense from a distance when passing by a house where kita is being baked from dabi teff flour.

One of the key informant interviewers, the DA said:

… when I go to the village to visit farmers, I know where dabi teff kita is being prepared. It always causes me a mouth‐watering stimulation to eat it because of the good flavor I sense….

Further, the participants described the preparation and consumption scheme of the food form, Cafaqoo, prepared from dabi teff. One of the FGD discussants elaborated:

… Cafaqoo is a small, rounded bolus‐like food, prepared by breaking fresh kita into smaller pieces and mixing it with some amount of refined butter. During Cafaqoo preparation, more salt is added than usual and it will be eaten more often as a dinner meal and it evokes a person to drink more water who has eaten it. Cafaqo is prepared with the addition of spices such as onion, garlic, black cumin and cardamom and was used as sinqi in a long time back when traders go a long distance for marketing (more than a week time walk on foot)…

In addition to the different traditional food forms, home‐brewed traditional alcoholic drinks could also be prepared from dabi teff and used for income generation. One of the 52 years of FGD female participants described:

… I have been preparing alcoholic beverages such as tella (local opaque beer) and katikala (local spirit) by using dabi teff as an ingredient for a long period in my life and I have been earning money by selling it. The tella and katikala from dabi teff is even very strong…

The development of nutritious complementary foods from locally available and underutilized food crops has received a lot of attention in low‐income countries. The participants were asked whether they used dabi teff for the preparation of child food and it was responded that it is not common.

Most of the participants responded that as follows:

…the use of dabi teff as a complementary food is not common. We usually use other crops such as barley, maize, oats, and chickpeas for the preparation of complementary food for our children. We mostly use dabi teff for the nutritional treatment of sick, delivered women and bone‐fractured persons and we often reserve it for such events. But rarely, some people might add as an ingredient for weaning food preparation…

One Key informant interviewer suggested as follows:

… even though it is not common practice to prepare complementary food from dabi teff, there is no foreseen problem if it can be used as a complementary food, even since it is rich in iron content, it would be good food for the children's development, it would increase their blood volume….

### 3.3 Social Beliefs Linked to Dabi Teff in the Community

Almost all the participants shared common beliefs linked to dabi teff in the community. Most of the social beliefs were associated with the consumption of the different food forms that were described earlier. These beliefs were composed under energy value, medicinal value, and workforce efficiency.

The beliefs or the lived experience associated with the consumption of mooqa manyee was described by one of the FGD participants, where it was said as follows:

…a person who ate mooqa manyee became stronger and braver the next day. A male who has eaten such food form shouldn't approach a female for a sexual act because it is believed that he might harm her. So he shouldn't approach her by any means on the day he has eaten it. He can approach the next day. It is also used as a medicinal food….

The medicinal values of mooqa manyee were potentiated by one key informant by verifying its health values that result in positive health and nutritional outcomes when served to the member of the family who needs special nutritional treatments, the food therapy. One elder female in the FGD where her idea was also supported by a key informant interviewer stated as follows:

… when mooqa manyee is served to a recently delivered mother, she would recover from her backbone pain shortly and fast weight gain (postnatal nutrition) and if served to a bone‐fractured person he/she would heal fast, it is also used for the nutritional treatment of sick people, with the actual observed health outcome ….

The beliefs were further supported by the female FGD participants, who said as follows:

… after eating the different food forms, particularly the mooqa manyee and cafaqoo, it increases blood volume, boosts energy/strength, and repairs/strengthens the backbone and fractured bones …

A 54‐year‐old female FGD participant raised the improvement of workforce efficiency following the consumption of mooqa manyee and cafaqoo, and she stated as follows:

… Mooqa manyee and Cafaqoo are usually prepared for males who are going to plow or till farmland because they will not get tired and/or feel hungry the whole day. It gives males more energy to work hard without tiring …

### 3.4 Trends of Dabi Teff Cultivation (From Past to Present)

There is an old popular traditional saying, daabiin angafa midhaaniiti, meaning dabi teff is a leading crop of all cereals, known among the Oromo people in western Ethiopia. All of the discussants (the FGD and key informants) have noted that they are very familiar with dabi teff and it has been cultivated in their area since ancient times. Dabi teff produce can either be used for home consumption or for earning money by selling it in the market. It is still commonly cultivated by many farmers in the area and much praised among the community, but most of them are arguing that its cultivation is declining nowadays and they fear that its cultivation will be forgotten in the long run.

One of the male FGD participants stated that:

… Dabi teff is well known in our area and it is a historical crop that people's minds are associated with the golden quality of this crop. Mostly, we use dabi teff grain for home consumption or sell it to earn money. He further stated that its cultivation pattern from the past to the present is a bit declining. He added, when we think of dabi teff cultivation, mostly its traditional and medicinal value comes into our mind, which is less considered among the younger people…

Another FGD participant supported this idea and mentioned as follows:

… yes, dabi teff has been cultivated since ancient times and our community has a special opinion/perception for dabi teff, they just see it as a medicinal food and give special respect for the food forms prepared from it…

Further, the participants from the key informants stated as follows:

… if we have dabi teff crop in our home, we feel like we have medicine at home. Most of the time, it is reserved for “the time of problem” to be used as treating of a family member who needs special nutritional treatment, such as by preparing its gruel and serving it to a delivered woman and sick people…

### 3.5 Contribution of Dabi Teff in Addressing Food Security

Between June and early August, households in most parts of Ethiopia are in a food crisis every year. All the participants (FGD and key informants) have described that dabi teff reaches maturity in a short time for harvest since its cultivation. Thus, they appreciated the contribution of dabi teff in ensuring their food security, particularly during the summer season when there is a food shortage in most households. One male FGD participant said as follows:

… due to the very short maturity period of dabi teff, it is harvested twice within one rainy season (1st, during the onset of Kiremt rainfall, called “daabi gannoo” & 2nd late rainfall after mid‐September, called “daabi birraa”). We usually call dabi teff “Ji'a‐Lame” to mean a crop reaches harvest in two months as it usually reaches harvest in 70‐80 days…

This early maturing and less rainfall requirement make dabi teff a unique variety teff amenable to climate‐smart agriculture. One of the participants said as follows:

… in addition to cultivating dabi teff on the usual farmland by application of fertilizers, it can also be cultivable on irrigation land during the winter time….

### 3.6 Thrashing System of Dabi Teff

Dabi teff threshing is carried out by squashing over the cut crop collected on a flat surface and driving cattle (oxen) over it. It can also be thrashed by using a special wooden stick. A 62‐year‐old FGD participant stated as follows:

… before trashing dabi teff, we habitually prepare a flat surface in the appropriate place and seal it using fresh cow dung. Women perform the sealing activity and they mix the cow dung with water (thickly mixed) and it would be slowly dried so that the cow dung gets epoxied to the flat surface. He mentioned, this is done to ensure that it would not get cracked and to prevent seed loss during the threshing. To avoid the seed from soil contamination or to prevent any soil from entering into the sealed floor, the women create a kind of embankment (dike) surrounding all the sealed floor, called timjjesssu in afaan oromoo. After it gets dried well, the women check it and clean the dried sealed floor surface using a cleaner called dry harata to make it a very clean surface with no cow dung debris that can contaminate the seeds. The women do the cleaning in the late afternoon of the previous day to trash it in the late morning (from 10 to 11 O'clock) of the next day using wooden sticks….

The trashing system was substantiated by another FGD participant who said as follows:

… to trash dabi teff, we use a special wooden stick which is prepared technically by bending one end of the wooden stick looking like a fork and when we beat, we would not beat directly down to the floor, but it has its own beating fashion (inclined beating) that only expertise can perform it. This is particularly to avoid cracking of the sealed flour, thereby avoiding soil contamination and seed loss….

**FIGURE 3:**
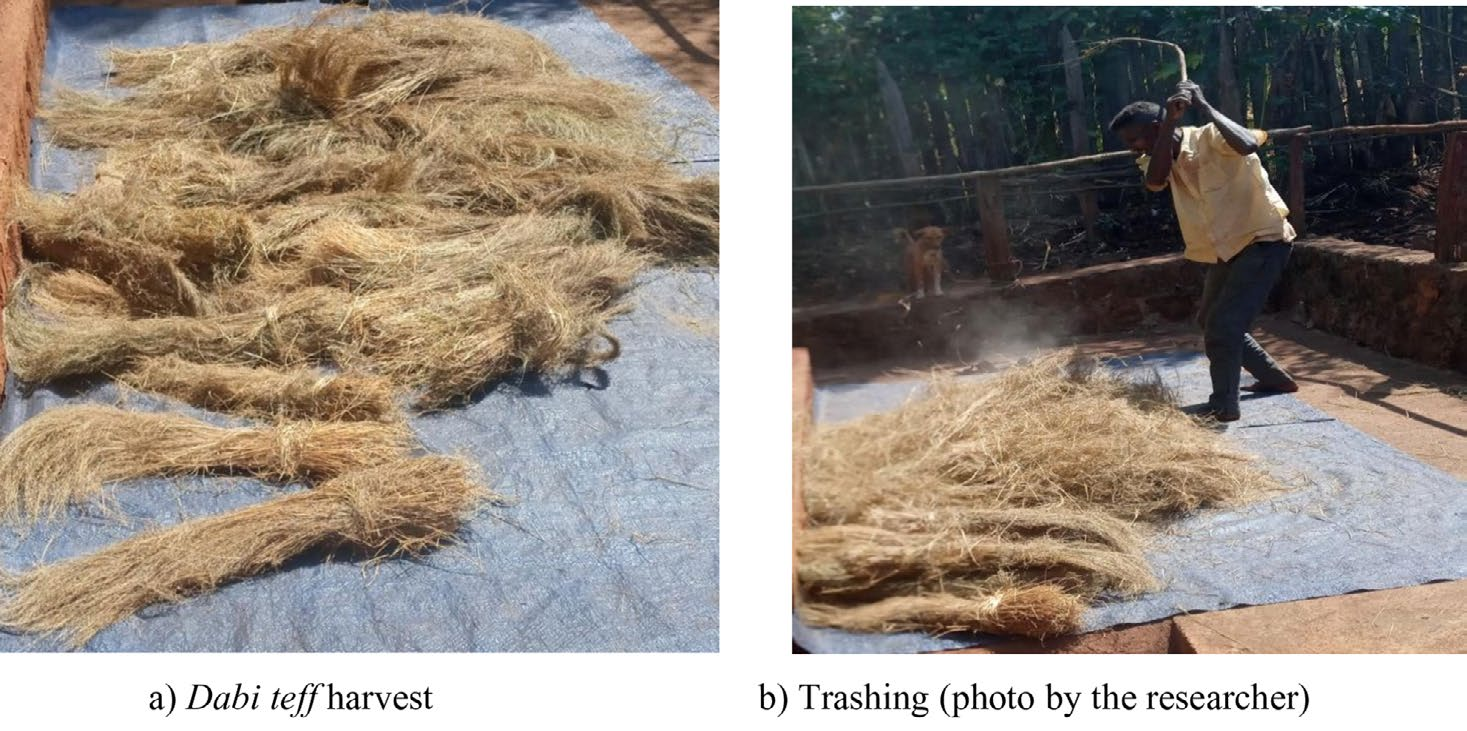
Dabi teff harvest and trashing system. (a) Dabi teff harvest; (b) trashing.

The same idea was again supported by another key informant's interviewer, mentioning as follows:

… in case any trash ground floor cracking is observed, the women would re‐seal it and it has to be waited for one day to till drying. Now time people have also started to use what we call ‘Shara’, which they place it on the flattened surface…. (Figure 3).

### 3.7 Separation of the Grain From Its Thrash (Chaffs or “Galaba/Chid”)

**FIGURE 4:**
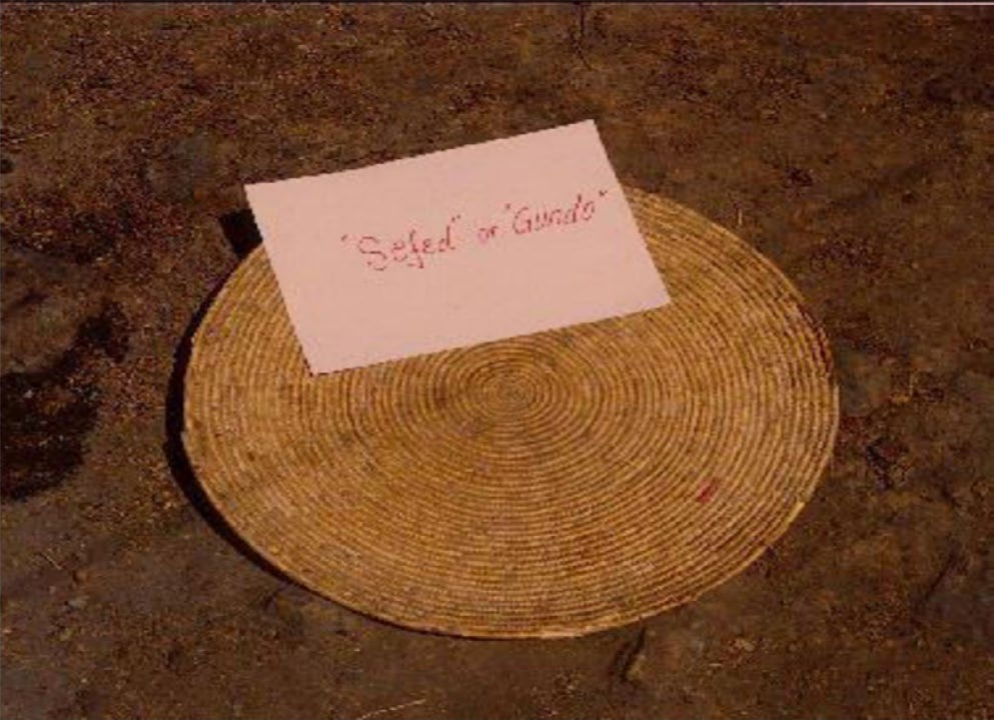
Gundoo or Sefed (photo by the researcher).

**FIGURE 5:**
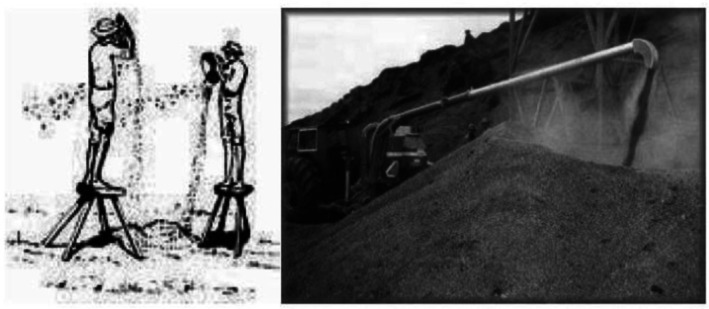
Small and medium‐level teff grain cleaning.

First, the straw would be removed using the beating stick, and the separation of teff grain from its chaff is carried out by throwing the trashed grain high into the air, following the difference in aerodynamic or air‐bellowing properties. During the separation of the threshed materials, the fundamental forces involved are the weight/gravity of the seeds and the aerodynamic wind drag. One key informant interviewer stated as follows:

… to separate the thrashed material from the seed, we use a material called sefed/gundoo (Figure 4), a kind of fan woven prepared from a grass stem. This operation is carried out by lifting the fan woven above our height or standing on a wooden footstool and letting it drop smoothly so that the blowing wind will separate the seeds from the chaff by following the wind direction (Figure 5). The winnowing has its own technique in that we see the direction of the wind and we move around to adjust ourselves to the direction the wind below. If the wind speed is stronger, we stop it for a while until the wind calms down and becomes moderate. Unless the seed is blown away because of its smaller weight. The remaining inert materials are removed by fanning with a material called Afarssa/Maragabiya (small sefed or stiff dried cattle skin)….

Another participant supported the same idea by stating as follows:

…If there is no wind bellowing at all, there is a common traditional practice that males whistle‐out believing that the wind would come and it usually happens. It is a kind of ordering nature (wind) to obey the whistle sound….

### 3.8 Storage Quality of Dabi Teff

Dabi teff seed could stay for a long time after harvest if properly stored. If it is stored in a dry environment, it can stay for a longer period without being damaged by pests and spoilage.

This was explained by a male FGD participant as follows:

… Dabi teff grain has good storage quality so it will not be damaged by any pests and it can stay for many years, up to 4 to 5 years, even more without damage if stored under normal conditions. They call such dabi teff ‘Daabi dilbii’ to mean the dabi teff that was stored and stayed for long times after harvest. He stated that such a type is more preferred for ‘Muk’ (gruel) preparation….

### 3.9 Contribution to House Construction

The discussants were further probed for any other use of dabi teff, and all the participants (FGD and key informants) expressed that, on top of its seed harvest for consumption and medicinal importance, the trashed straw is composed of fine stems used for plastering (finishing) mud hut walls, mud silo making, and contributes to house construction.

Most FGD participants said as follows:

… when we will have a plan to construct a house in the coming year, we cultivate dabi teff ahead, carefully collect and reserve the straw of dabi teff for using it for plastering mud hut walls. This is because the straw is very fine and it cements the mud together, preventing it from cracking….

### 3.10 Contribution for Animal Forage

The participants, when they are expressing the additional values of dabi teff, have stated the forage potential of dabi teff straw. After trashing, they accumulate the straw and store it in a safe, dry place. Most of the discussants describe as follows:

… the value of dabi teff is not only the seed, but the straw is also very useful for animal feeding. We store the straw in a safe place till summer time comes to feed our cattle. It is specially used for feeding bulls or oxen after plowing or tilling farmland because it is believed that the oxen would become strong. We also use it for feeding other animals like milking cows to provide more milk and horses…

One male FGD participant stated the following, substantiating this opinion:

…. during the past times, we also mixed dabi teff and finger millet flours to prepare leavened bread by adding some salt to it and used to feed oxen. It is believed that when the ox is fed such kind of food, it would get more strength and we don't observe a feeling of tiredness from the ox when tilling land….

## 4 Discussion

Dabi teff is a marginally cultivated and/or underutilized food crop grown in Western Ethiopia. The result of this study showed that there are many sociocultural values, traditional usages, social beliefs, and cultivation trends associated with dabi teff among the communities in the study area. In‐home consumption, dabi teff can be used for the preparation of important traditional healthy foods such as injera/budena (pancake‐like local bread), kitta/maxino/cacabsa (unleavened bread), atmit or muk/mooqa (gruel), mooqa manyee, fetfet/fafato, cumboo, cafaqoo, and cacabssa (the favorite cultural dishes) and bread for all age groups, including children in Ethiopia (Baye et al. 2012; Temesgen 2013). Another traditional usage of dabi teff is the preparation of homemade local brews or local alcoholic drinks such as tela/farsoo and katikala/arakee that the women use for income generation.

This study has generated real information/knowledge from the community, which is crucially important to design appropriate food‐based nutrition intervention strategies. A food belief study allows for the identification of positive and negative social cognitive factors that may influence both the production and consumption of a crop. The FAO and WHO (2006) recommend the collection of detailed baseline information on traditional practices and food beliefs prior to designing and initiating food‐based intervention programs.

In addition to the enjoyment of many traditional food forms of dabi teff, the result shows that there are also many social beliefs associated with dabi teff. The participants reflected many health and nutritional benefits following eating the different food forms, particularly the mooqa manyee and cafaqoo, which increase blood volume, “dhiiga dabalaa” boosts energy/strength, “humna dabalaa” and repairs/strengthens the backbone and fractured bones, “dugda jabeessa”.

The findings from this study showed that dabi teff grain matures in less than 90 days, due to which it is harvested twice within one rainy season, during the onset of Kiremt rainfall, late rainfall after mid‐September; thus, it allows a good double cropping option following the harvest of crops such as maize in late September. Between June and August, households in most parts of Ethiopia are in a food crisis every year (NET and WFP 2018). However, households in West Ethiopia address their food security problem during such food shortage times by cultivating dabi teff at the early onset of Kiremt rainfall, showing its potential in responding to the food insecurity challenge.

Despite the aforementioned traditional usage (the preparation and consumption schemes), social beliefs, nutritional and health benefits of dabi teff, there are various shortcomings in its production. Some of the major ones are: low productivity, laborious production from its cultivation till trashing, its need for commercial fertilizers and overemphasizing other stable crops such as maize, sorghum, and barley (Daba 2017). Further, the young population is showing ignorance of taking the traditional practices and passing them down to the next generation. Therefore, the chances of dabi teff continuing as highly liked and praised among the community in western Ethiopia are doubtful because traditional knowledge linked with dabi teff is in danger of eroding and disappearing.

This study revealed that the threshing of dabi teff and its separation from the chaff is carried out under very controlled conditions, such that the contamination of its seed with the soil is almost none. Some scholars think that the high iron content of teff is due to contamination from iron‐rich soil ground on the external surface of the grains. However, a higher value was obtained using fresh teff from the plant, threshed in the laboratory (Daba 2017; Tura et al. 2023a). Actually, the iron content of teff might vary depending on the crop variety and soil conditions, but still, it contains a high iron content wherever sampled (Daba 2017; Tura et al. 2023a).

Some others claim that the iron is actually embedded in the grain walls and considered a dietary source of iron along with the actual true iron content of the grain itself (Daba 2017). Despite the arguments, it has been proven that teff grain has a high iron composition. One point is that not all soils of teff‐growing areas are ferruginous, and the other point is that only a small portion of the bulk teff is contaminated with soil, even in the traditional threshing style. The threshing grounds are made with care to avoid soil contamination by sealing with an animal dung and teff straw mixture. In research stations, teff is harvested and threshed with care, not on the ground; hence, there is a low chance of soil contamination. Still, teff is reported to have high iron content (Daba 2017). Dabi teff straw is composed of fine stems used mainly for reinforcing mud for plastering wooden walls of buildings. The straw is stored and used as an important livestock feed, especially during the dry season (Ketema 1997). Cattle prefer teff straw to straw from any other cereal, and its price is higher than that of other cereals. Hence, teff crops, including dabi teff, can be used as a dual or multipurpose crop, that is, both as a cereal food and as forage feed (Ketema 1993).

One of the findings from this study was the storage quality of dabi teff. Thus, it has a high economic value as its grain can be kept for many years in practice when kept in any kind of storage facility without being seriously damaged by common storage insect pests. Some farmers store maize and other pulse crops along with dabi teff. This is due to the minute size of the dabi teff seeds, which could hamper the movement of weevils inside the seeds, and due to the low level of oxygen, by closing air holes, insects cannot grow inside and damage the crops (Dijkstra et al. 2008). Furthermore, the low level of oxygen might not allow fungal growth and mycotoxin production, which accounts for the good storage quality of dabi teff (Dijkstra et al. 2008). In summary, we found out that dabi teff is primarily worth as a traditional food, medicinal food, and a good energy source, and one of the highly valued food commodities in western Ethiopia.

The important finding from this study was that, even though the community has strong traditional practices and connection with dabi teff, there was less practice of using it as a complementary food, not only in the study area but also, there is less practice of using red teff as a child food in the general population in Ethiopia which could be the reason for the prevalence of a higher rate of anemia (57%) in under‐five children (EDHS 2016).

WHO (2003) recommends the use of nutritious indigenous or staple foods together with traditional processing technologies such as fermentation and germination, among others, to produce nutrient and energy‐dense complementary foods that need to be promoted. We, the researchers, suggested that enriching child food through incorporating dabi teff flour might be nutritionally promising, which could be used as a nutrition intervention to address both the protein‐energy and micronutrient malnutrition for optimal growth and development of children.

## 5 Conclusions

This study presented culturally significant ethnographic information documenting traditional knowledge and practices surrounding dabi teff, an ancient indigenous underutilized crop widely grown in Western Ethiopia. There are many sociocultural values, as well as recognized nutritional and health benefits of the crop among the community. Particularly, when mooqa maynee, a typical food form from dabi teff, is served to a delivered mother and/or bone‐fractured person, it results in proven nutritional and health outcomes. The study has shown that dabi teff reaches maturity in a short time and has good storage quality, where the crop could play a substantial role in ensuring food security. Use of the crop as a complementary food is not common. There is ignorance in accepting and passing down the traditional practices due to which dabi teff cultivation is declining nowadays, and there is a fear that all those sociocultural values would be forgotten in the long run. Thus, it is suggested that dabi teff cultivation needs to be integrated into Ethiopian agricultural programs and into national nutrition strategies. Additionally, incorporation of the findings into child complementary feeding programs, as well as into agrobiodiversity conservation initiatives, are strong recommendations to translate the study into policy.

## Author Contributions

**Diriba Chewaka Tura:** conceptualization (lead), data curation (lead), formal analysis (lead), investigation (lead), methodology (lead), project administration (lead), resources (lead), software (lead), supervision (lead), validation (lead), visualization (lead), writing – original draft (lead), writing – review and editing (lead).

## Conflicts of Interest

The author declares no conflicts of interest.

## Supporting information


**Appendix S1:** fsn371130‐sup‐0001‐AppendixS1.docx.

**Appendix S2:** fsn371130‐sup‐0002‐AppendixS2.docx.

## Data Availability

All supportive data are contained within the article in tables, figures and Appendices S1 and S2.
