# Supplementary material for: Ethnographic Qualitative Study to Explore the Sociocultural Values, Nutritional Potential, and Health Benefits of Dabi Teff ( Eragrostis Tef ) Grown in Western Ethiopia
Source: Food Sci Nutr. 2025 Oct 25;13(11):e71130. doi: 10.1002/fsn3.71130 (PMC12552782; doi:10.1002/fsn3.71130)
Supplement: Supplementary file 1 — Appendix S1: fsn371130‐sup‐0001‐AppendixS1.docx. [file FSN3-13-e71130-s001.docx]

**Key Question guide for Ethnography focus group discussion (FGD) regarding *dabi teff*.**

| Semi-structured questionnaire | Expected outcome |
| --- | --- |
| 1. What are the preparation and consumption   schemes of *dabi teff* (traditional usages)? | The different food forms that can be prepared from *dabi teff* and the eating pattern. |
| 2. What are the nutritional and health benefits   associated with *dabi teff*? | Knowledge of the role of healthy foods in nutritional treatment. |
| 3. What are some social beliefs or claims   linked to *dabi teff* | To generate social beliefs or claims rooted in the community regarding *dabi teff*. |
| 4. How is the cultivation trend of *dabi teff* in   the area? | The pattern of cultivation trend from the past to current. |
| 5. In what duration does *dabi teff* reach for   harvest? | The number of days *dabi teff* reaches for harvest. Its role to ensure food security in harsh times. |
| 6. What type of crops do you use for the preparation of complementary food in this area? | Identifying the locally available crops for complementary food preparation. |
| 7. Do you use *dabi teff* for the preparation of complementary food? | The practice of using *dabi teff* as complementary food |
| 8. Other than its seeds, is there any purpose of   cultivating *dabi teff* ? Probe. | Multi-purpose of *dabi teff*. |
| 9. How is the trashing and cleaning system of   *dabi teff* seeds? | Understand the possible contamination with soil. |
| 10. For how long can you store *dabi teff* without   damage? | Storage quality without deterioration |
| 11. Any other benefits of *dabi teff* cultivation?   Probe and take note. | Other contributions of *dabi teff* cultivation beyond food purpose |
